# Exploration of the roles of microbiota on biogenic amines formation during traditional fermentation of *Scomber japonicus*

**DOI:** 10.3389/fmicb.2022.1030789

**Published:** 2022-11-02

**Authors:** Jingyi Chen, Haiqing Tang, Mengsi Zhang, Shangyuan Sang, Lingling Jia, Changrong Ou

**Affiliations:** ^1^College of Food and Pharmaceutical Sciences, Ningbo University, Ningbo, China; ^2^Faculty of Food Science, Zhejiang Pharmaceutical University, Ningbo, China; ^3^Key Laboratory of Animal Protein Food Deep Processing Technology of Zhejiang Province, Ningbo University, Ningbo, China

**Keywords:** fermented *Scomber japonicus* (*zaoyu*), biogenic amines, MiSeq sequencing, microbiota composition, PICRUSt

## Abstract

The influence of microbiota composition and metabolisms on the safety and quality of fermented fish products is attracting increasing attention. In this study, the total viable count (TVC), pH, total volatile base nitrogen (TVB-N) as well as biogenic amines (BAs) of traditional fermented *Scomber japonicus* (*zaoyu*) were quantitatively determined. To comprehend microbial community variation and predict their functions during fermentation, 16S rRNA-based high-throughput sequencing (HTS) and phylogenetic investigation of communities by reconstruction of unobserved states (PICRUSt) were employed, respectively. The fresh samples stored without fermentation were used as controls. TVC and TVB-N values increased rapidly, and the content of BAs exceeded the permissible limit on day 2 in the controls, indicating serious spoilage of the fish. In contrast, a slower increase in TVC and TVB-N was observed and the content of BAs was within the acceptable limit throughout the fermentation of *zaoyu*. Significant differences in microbiota composition were observed between *zaoyu* and the controls. The bacterial community composition of *zaoyu* was relatively simple and *Lactobacillus* was identified as the dominant microbial group. The accumulation of histamine was inhibited in *zaoyu,* which was positively correlated with the relative abundance of *Vibrio*, *Enterobacter*, *Macrococcus*, *Weissella*, et al. based on Redundancy analysis (RDA), while *Lactobacillus* showed a positive correlation with tyramine, cadaverine, and putrescine. Functional predictions, based on Kyoto Encyclopedia of Genes and Genomes (KEGG) pathways analysis, revealed that the relative abundance of metabolic function exhibited a decreasing trend with prolonged fermentation time and the abundance of metabolism-related genes was relatively stable in the later stage of fermentation. Those metabolisms related to the formation of BAs like histidine metabolism and arginine metabolism were inhibited in *zaoyu*. This study has accompanied microbiota analysis and functional metabolism with the accumulation of BAs to trace their correspondences, clarifying the roles of microorganisms in the inhibition of BAs during fermentation of *Scomber japonicus.*

## Introduction

*Scomber japonicus* is an economically important fish species extensively distributed in the East China Sea, the Yellow Sea, and the Sea of Japan (Yu et al., 2018). It is noted for its pleasant aroma and high nutritional value, whereas it is rich in free histidine in muscle tissues, which is commonly implicated in incidents of histamine poisoning (Visciano et al., 2012). Hence the preservation of *Scomber* has been a quite challenging issue. Fermentation plays a vital role around the world for the preservation of aquatic products, such as *Narezushi* in Japan (Doi et al., 2021), *Gravlax* in Northern Europe (Wiernasz et al., 2020) and *zaoyu* in China. *Zaoyu* is a traditional fermented product made by mixing fish and fermented rice, then sealed in the vessel for a long-term fermentation under anaerobic conditions, which is usually made from several marine species such as *Scomber japonicus, Miichthys miiuy, Trichiurus lepturus, Muraenesox cinereus*, and freshwater species (Chen et al., 2021). The fermentation process imparts a distinctive flavor to the final products. Typically high levels of biogenic amines (BAs) have been found in some fermented fish products like fish sauce (Kuda et al., 2012) and dried fish (Huang et al., 2010), owing to the availability of amino acids, which may be the potential precursors of BAs. However, it’s worth noting that the formation of BAs was inhibited during the preparation of *zaoyu*.

BAs are low molecular weight organic bases with biological activity that are formed by microbial decarboxylase of the corresponding amino acid or transamination of aldehydes and ketones by amino acid transaminases (Zhai et al., 2012). The intake of foods with high concentration of BAs could provoke those adverse reactions like migraine, brain hemorrhage, heart failure, hypertension, urticaria, and headache as well (Rice et al., 1976). The most common sources of BAs intoxication are histamine (His) and tyramine (Tyr) (Smith, 1981), produced by the decarboxylation of histidine and tyrosine, respectively. Putrescine (Put) and cadaverine (Cad) could enhance histamine toxicity through interfering with the histamine detoxification system (Kim et al., 2009). The formation of BAs in fermented foods might result from a complex process and could be influenced by many factors and their interactions (Sang et al., 2020).

Spontaneously fermented fish has a unique flavor, while it is restricted by the relatively long production period and the risk of spoilage is higher. Hence the inoculum of suitable microorganisms, defined as starter cultures, has become a widely adopted approach in fermented food. Bover-Cid et al. have shown that the incorporation of pure or mixed amine-negative starter cultures is capable of inhibiting microorganisms with amino acid-decarboxylase activity in fermented products (Bover-Cid et al., 2000; Hu et al., 2007). However, the use of commercial starter cultures may reduce the microbial diversity of fermented food, and the dominant bacteria may inhibit the growth of other microorganisms including those contributing to the flavor formation. *Zaoyu* is produced on the basis of the traditional fermentation method, mostly relying on the community of autochthonous microorganisms from fermented rice. The addition of fermented rice as natural starter culture differs from spontaneous fermentation, which could not only promote the appearance of dominant bacteria, but also be used as the supplementary substrate for carbon sources during traditional fermentation (Bassi et al., 2015). Besides, figuring out the microbiota composition of *zaoyu* is vital for the application of synthetic microbial community (SMC) in fermented foods, which could construct a controllable and stable microbial interaction network and might serve as a potential strategy to control the quality of fermented fish in the future (Han et al., 2022).

Dynamic changes and interactions of the microbiota during fermentation were found to play a critical role in the quality characterization. Since spoilage potentials and metabolic characteristics of different microbes vary significantly and could be affected by microbial interactions (Lu et al., 2018). High-throughput sequencing (HTS) with the properties of high flux and short experimental cycle has recently been applied to the analysis of microbial systems in different food matrices, such as fish sauce (Wang et al., 2018), reef fishes (Gao et al., 2020), and Pacific white shrimp (Guan et al., 2021). A great potential of HTS is facilitating the exploration of the relationships between the microbiota composition and specific variables like BAs (Hao and Sun, 2020), salinity (Mengjuan et al., 2021). Phylogenetic investigation of communities by reconstruction of unobserved states (PICRUSt), a technique predicting metagenomes based on 16S rRNA gene data and a reference genome database (Yan et al., 2019), provides a great amount of information about the genetic profile and metabolic potential of microbiota composition. Combined with both technologies could determine the dynamic changes of microbial community in *zaoyu* and get a comprehensive view of the role that fermentation played in inhibiting the accumulation of BAs from the perspective of microorganism.

The aim of this study is to explore microbial succession during fermentation of *Scomber japonicus* and demonstrate the correlation among the microbiota composition, functional metabolism and the formation of BAs. The microbiota composition of *zaoyu* also may serve as a promising model system to study eco-evolutionary dynamics, such multidisciplinary approach is expected to improve the property and quality of fermented foods.

## Materials and methods

### Source of samples

Fresh *Scomber japonicus* was obtained from a local market in Ningbo, Zhejiang Province, China, and filleted to collect the dorsal muscle. The dorsal muscle was cut with a knife into blocks of 7 × 7 × 4 cm and were cleaned with tap water and mixed with 12% saline marinate in a barrel for 3 h, then drained the water for 12 h. *Zaoyu* was prepared according to the traditional techniques by mixing fresh fish with fermented rice and were neatly stacked layer-by-layer in a barrel for fermentation. Then *Zaoyu* samples were collected at 0, 1, 2, 4, 6 and 8 days of the fermentation stages for further analysis as experimental group. The fresh *Scomber japonicus* stored at 28°C under the same conditions were used as controls, and fermented rice was collected as the other controls named Z. All of the experiments were conducted in three replications.

### Methods

#### Enumeration of cultivable microbes

The TVC was determined based on the National Standards of the People’s Republic of China (GB 4789.2-2016). Briefly, an aliquot of 2.5g fish filets was collected in an aseptic manner, put into aseptic bag, and homogenized in 22.5 ml of physiological saline for 2 min. The homogenates were serially diluted with sterile physiological saline (1:10), and 1.0 ml aliquots of the dilutes were poured onto a 15–20 ml of plate count agar medium to obtain a mixture. Colonies on the plates were identified posterior to incubation under 30°C for 72 h. The total number of the colony was described as lg CFU/g.

#### Determination of physicochemical indices.

The water content was measured with a moisture analyzer (MB27; OHAUS, American). An aliquot of 2.0 g samples was homogenized in 20 ml of distilled water at room temperature and then recorded *via* a digital pH meter (PHS-2F, Leici, Shanghai, China). TVB-N levels were identified *via* a semi-micro determination of nitrogen approach as per the Chinese Standard (GB 5009.288-2016). All samples for analysis were ground individually using a meat grinder. An aliquot of 4.0 g ground filets was taken into a beaker, blended with 20 ml distilled water, then impregnated still for 30 min and shook the beaker every 10 min. Next, the solution was filtered through the filter paper, then 5 ml of filtrate was made alkaline by adding 5 ml of 10 g/L Magnesia (MgO). Steam distillation was distilled for 5 min using a Kjeldahl distillation unit. The distillate was absorbed by 10 ml of 20 g/L boric acid, and titrated with 0.01 mol/L HCl. The result stated for each sample is the mean value of two measurements, TVB-N content was calculated and expressed with a unit of mg/100 g.

#### Determination of biogenic amines

The separation and quantification of BAs were carried out according to the liquid chromatography–tandem mass spectrometry method (Waters Alliance e2695) using 1,7-diaminoheptane as internal standard. For the analysis, 2 g of sample was ground in a Waring blender for 60 s and thoroughly homogenized with 20 ml of distilled water. The obtained homogenate was decanted into centrifuge tubes and added acetonitrile (20 ml), ethanol (3 ml), n-hexane (5 ml), followed by centrifugation (5,000 rpm, 5 min). The supernatant was collected and cleaned with methylene chloride (2 × 2 ml) twice. The solution was then filtered with 0.45 μm filter paper and diluted 1:4 with pure water before injection in the column equipped with ACQUITY HSS T3 (100 mm × 2.1 mm, 1.8 μm; Waters Technologies (Shanghai) Limited). The mobile phases of LC–MS/MS were A: 2 mM/L ammonium acetate, and B: methanol containing 0.1% formic acid. The flow rate was 0.2 ml/min.

#### Total DNA extraction, PCR reaction and MiSeq sequencing

Total genomic DNA was extracted from the muscle of mackerel samples (10 g) with the instructions of the E.Z.N.A^®^ Bacterial DNA kit following the manufacturer’s instructions. DNA samples were then stored at −80°C before amplification. The V3-V4 (341F: 5′-CCTACGGGNGGCWGCAG-3′, 806R: 5′-GGACTACNNGGGTATCTAAT-3′) hypervariable regions of the bacterial 16S rRNA genes were, respectively, amplified using the genomic DNA extracts for each sample. The reactions mixture (25 μl) included 2.5 μl of 10 × PCR buffer, 2.0 μl of dNTP, 0.5 μl of each primer, 1.0 μl of DNA, 0.5 μl of TaqE and 18 μl of ddH_2_O. PCR reactions were conducted in a Thermal Cycler under the following conditions: initial denaturation at 95°C for 3 min, followed by 30 cycles of denaturation at 95°C for 30 s, primer annealing at 57°C for 1 min, and extension at 72°C for 45 s, and a final extension step at 72°C for 10 min. The PCR products and expected size were checked by 1% agarose gel electrophoresis and were purified with a quick gel extraction kit. The concentration and quality of amplicons were measured using a NanoDrop-1000 spectrophotometer. Sequencing was performed on an Illumina MiSeq platform at Novogene Bioinformatics Technology Co. Ltd.

#### Statistical analysis

The bioinformatics analysis was performed with QIIME 2 (2021.11). Then quality filter, denoising, merging, and chimera removal were conducted by the DADA2 plugin. The sequences were clustered at the level of 97% similarity and the representative sequence of each operational taxonomic unit (OTU) was annotated by Silva database. Analysis of α-diversity and β-diversity was completed based on the platform of Personalbio.^1^ The Linear discriminant analysis Effect Size (LEfSe) tool^2^ was used to analyze the different species among the sample groups. Phylogenetic investigation of communities by reconstruction of unobserved states (PICRUSt) software and database was used to obtain Kyoto Encyclopedia of Genes and Genomes (KEGG) pathway information. Redundancy analysis (RDA) on interrelationships between microbiota composition and BAs was analyzed by canoco5. Physiochemical properties of samples were analyzed, expressed on mean ± standard deviation. One-way analysis of variance (ANOVA) with Tukey pairwise comparisons at *p* < 0.05 was used to assess the significance between alpha diversity indices and relative abundance rates among each group using SPSS16.0.

## Results

### Microbiological and chemical analysis

**Figure 1:**
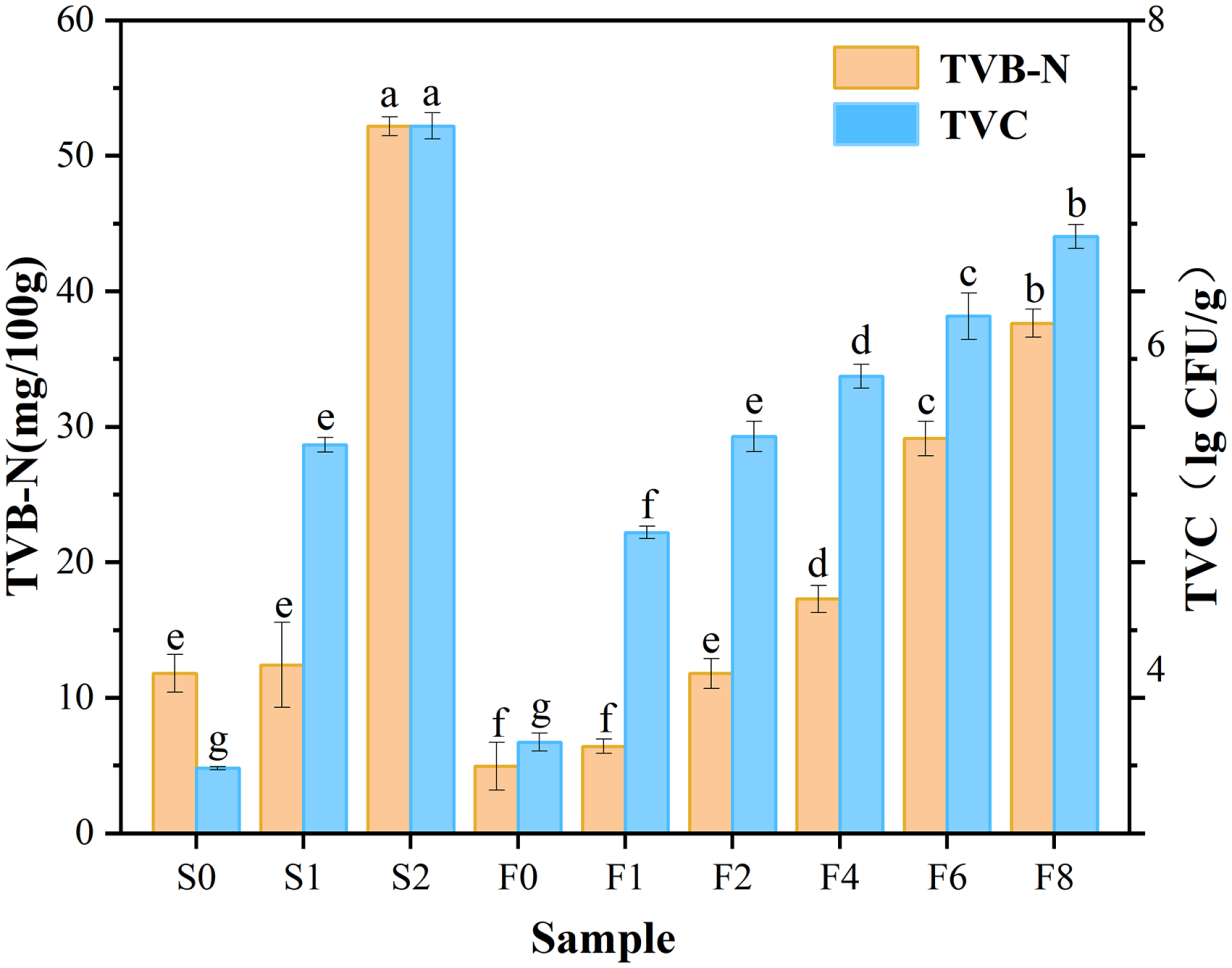
Changes in TVB-N and TVC values. Notes: S0, S1, S2 denote the controls on day 0, 1, 2, respectively; F0, F1, F2, F4, F6, F8 denote *zaoyu* on day 0, 1, 2, 4, 6, 8, respectively; Z denotes the fermented rice. Different letters (a, b, c etc.) indicate significantly different means at *p* < 0.05 (analysis of variance; ANOVA). The same below.

As shown in Figure 1, an increase in TVB-N value was observed during both storage and fermentation, which was in agreement with the microbial load. TVC values in the controls displayed an obvious upward trend and exceeded 7.0 lg CFU/g on day 2, which is the largest value that marine species are suitable for human consumption proposed by the ICMSF (ICMSF, 1986), and this observation was consistent with the trend in TVB-N value. Owing to the degradation of amino acids by microorganisms, the initial TVB-N value increased from 11.79 mg/100 g to 52.18 mg/100 g on day 2 of storage, which exceeded the upper acceptability limit of TVB-N in marine species of 30 mg/100 g according to the Chinese Standard GB/T 2733-2015, indicating it was already in a seriously spoiled state. Whereas it was observed that the amount of TVB-N increased relatively slighter in *zaoyu*, and TVC increased at a slower rate, being 6.67 lg CFU/g on the 8th day of fermentation, According to the microbial analysis below, the dominant microorganism was identified as *Lactobacillus*, which meant that the fermentation inhibited the growth of spoilage microorganisms. The formation of nitrogenous compounds in aquatic products is closely correlated with microbial metabolism indeed (Yu et al., 2019). The lower TVB-N and TVC values may be attributed to that the growth of some microorganisms is partially inhibited by the roles of fermentation. In addition, salting could also be an effective operation for inactivating microbes in the initial fermentation stage (Han et al., 2004).

### Physicochemical features

**Figure 2:**
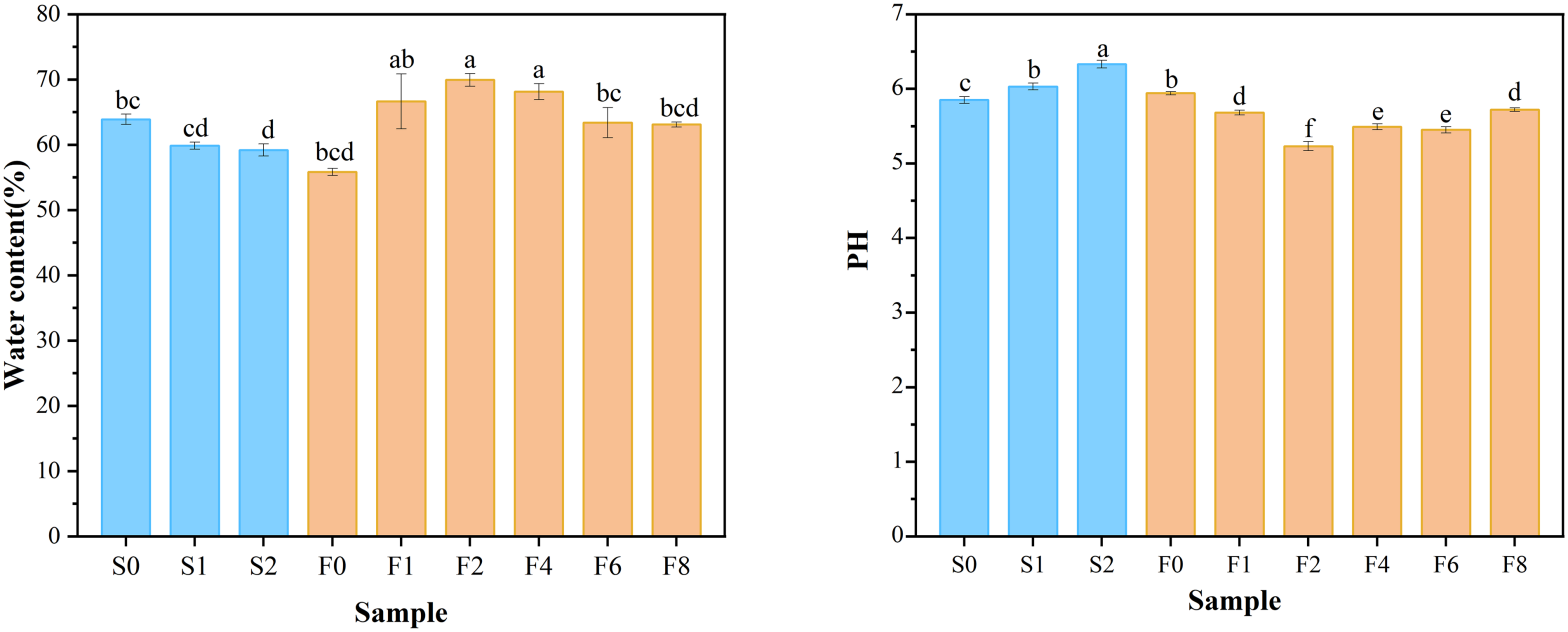
Variation in water content and pH.

The changes in moisture and pH were displayed in Figure 2. The increase in pH value during storage reflected the accumulation of TVB-N from bacterial activity and protein degradation (Moradi et al., 2019). In general, no significant changes in pH of *zaoyu* samples were observed. The initial pH presented a decreasing trend and reached a relatively stable value with slight fluctuation in the later period of fermentation. The decrease of pH was supposedly the result of the production of organic acids by fermentation (Jung et al., 2013), which was associated with the growth of *Lactobacillus.* The microbiota composition reached equilibrium in the late fermentation stage, which was also reflected in the pH changes. The slight increase in water content of *zaoyu* in the early stage may be attributed to the rehydration of the semi-dried fish soaked in the fermented rice, and the decrease on day 4 may be attributed to the changes in the water holding capacity of protein.

### BAs analysis

Totally 8 BAs were detected, including His, Tyr, Put, Cad, 2-Phenethylamine (2-Phe), spermidine (Spd), spermine (Spm) and tryptamine (Try), while octopamine (Oct) was not detected in any samples analyzed (Table 1). His, Cad and Put were the major amines in the spoilage of *Scomber japonicus*, among which His was the most dominant and increased rapidly, reaching 479.64 mg/kg on day 2 of storage, which far exceeded the limit level suggested by the U.S. Food and Drug Administration (FDA; 50 mg/kg). Compared with the controls, the content of His in *zaoyu* increased slightly and was below the allowable limit (50 mg/kg) suggested by FDA, indicating that the formation of His or the growth of microorganisms involved in the accumulation of His was inhibited during fermentation.

**Table 1:** Changes in the content of BAs.

Sample	Content of biogenic amines (mg/kg)								
	His	Cad	Put	Phe	Oct	Tyr	Spd	Try	Spm
S0	ND	ND	ND	ND	ND	0.06 ± 0.02^C^	1.57 ± 0.39^BC^	0.04 ± 0.02^D^	1.97 ± 0.59^A^
S1	145 ± 39.23^C^	10.42 ± 1.85^C^	ND	ND	ND	6.45 ± 2.77^C^	1.04 ± 0.16^CD^	0.03 ± 0.00^D^	2.28 ± 0.87^B^
S2	479.64 ± 46.95^B^	100.01 ± 5.92^B^	15.52 ± 2.18^D^	ND	ND	ND	0.65 ± 0.12^D^	0.03 ± 0.00^D^	2.10 ± 0.09^B^
F0	ND	ND	5.82 ± 0.09^D^	ND	ND	ND	1.44 ± 0.19^BC^	0.01 ± 0.00^D^	2.19 ± 0.33^B^
F1	ND	ND	8.40 ± 0.44^D^	ND	ND	26.53 ± 3.68^B^	2.26 ± 0.18^A^	2.18 ± 0.35^D^	2.07 ± 0.03^B^
F2	5.01 ± 0.16^A^	12.3 ± 0.66^C^	132.41 ± 3.28^C^	3.95 ± 0.15^C^	ND	87.91 ± 4.65^A^	1.7 ± 0.1^AB^	11.41 ± 0.94^C^	1.88 ± 0.06^B^
F4	0.95 ± 0.06^A^	8.47 ± 0.25^C^	134.3 ± 10.82^C^	3.90 ± 0.38^C^	ND	74.28 ± 4.53^C^	1.81 ± 0.45^AB^	10.76 ± 2.26^C^	1.73 ± 0.07^B^
F6	12.36 ± 0.64^A^	313.25 ± 24.96^A^	282.33 ± 23.62^B^	9.71 ± 1.04^B^	ND	99.01 ± 6.04^A^	1.88 ± 0.11^AB^	17.80 ± 1.39^B^	1.71 ± 0.04^B^
F8	4.38 ± 0.37^A^	287.58 ± 25.22^A^	342.99 ± 37.84^A^	14.21 ± 0.27^A^	ND	92.81 ± 6.93^A^	1.23 ± 0.03^BCD^	27.88 ± 0.43^A^	1.73 ± 0.00^B^

Values with different capital letters are significantly different (*p* < 0.05). S0, S1, S2 denote the controls on day 0, 1, 2, respectively; F0, F1, F2, F4, F6, F8 denote *zaoyu* on day 0, 1, 2, 4, 6, 8, respectively; Z denotes the fermented rice, and the same below. The same below.

Put, Cad and Tyr were relatively abundant BAs in *zaoyu* samples, and the content kept an upward trend during fermentation. Whereas the content of Tyr was still lower than the potentially dangerous level of 100–800 mg/kg (Elena et al., 2014), and neither legal limits nor toxic dose has been established for Put and Cad. A combination of Put and Cad has been suggested as an acceptable index in fresh meat, whereas it has proven not suitable to apply to fermented products (Ruiz-Capillas and Jiménez-Colmenero, 2004). The content of Spd and Spm in *zaoyu* was nearly unchanged, indicating that both BAs had negligible correlation with microorganisms. They are often considered to be physiological polyamines in organisms related to variety, location and physiological status (Önal, 2007). Zhai et al. have shown that Spm and Spd cannot be used as indicators for the evaluation of spoilage in fish fermentation (Zhai et al., 2012).

### Sequencing results and diversity indices

The number of reads per sample ranged from 16,154 to 36,342, 1,690 OTUs were obtained based on 97% similarity threshold. Alpha diversity indices were compared in different samples (Table 2). The coverage of sequences was over 0.99, showing that the data of amplicon sequencing with sufficient authenticity and depth. Shannon and Simpson are two main indices which comprehensively take the richness and evenness of microbial community of samples into account (Aregbe et al., 2019). In general, the four indices of bacterial communities presented a decreasing trend during fermentation, indicating that the richness of bacterial community decreased over time and a subset of bacteria became dominant in samples.

**Table 2:** Alpha diversity of bacterial community.

Sample	Shannon		Simpson		Chao1		ACE		Good coverage	
	Mean	SD	Mean	SD	Mean	SD	Mean	SD	Mean	SD
F0	3.77^c^	0.25	0.92^c^	0.02	388.97^f^	44.20	376.07	39.73	0.9983^a^	4.02E-04
F1	1.97^b^	0.29	0.73^b^	0.03	158.63^bc^	39.23	151.30^bc^	31.76	0.9995^cde^	1.59E-04
F2	2.13^b^	0.23	0.76^b^	0.03	115.44^ab^	21.57	115.64^ab^	21.19	0.9997^def^	1.17E-04
F4	1.45^a^	0.05	0.67^a^	4.70E-03	111.79^ab^	11.97	112.87^ab^	12.23	0.9998^def^	1.46E-04
F6	2.10^b^	0.07	0.76^b^	0.01	87.23^a^	9.12	87.55^a^	9.20	0.9998^ef^	1.06E-04
F8	1.52^a^	0.07	0.67^a^	0.01	61.61^a^	9.37	61.48^a^	9.10	0.9999^f^	6.22E-05
S0	3.89^c^	0.08	0.93^cd^	0.01	347.78^f^	30.96	344.86^f^	28.84	0.9991^bc^	2.63E-04
S1	3.89^c^	0.20	0.96^e^	0.01	224.28^de^	47.54	222.78^de^	52.55	0.9990^b^	2.28E-04
S2	3.81^c^	0.04	0.95^de^	3.85E-03	183.33^cd^	8.29	178.56^cd^	12.35	0.9992^bc^	1.79E-04
Z	3.98^c^	0.24	0.96^e^	0.01	252.82^e^	49.54	249.48^ef^	55.96	0.9994^cd^	1.52E-04

Values with different lowercase letters are significantly different (*p* < 0.05).

### Beta-diversity analysis of bacterial community

**Figure 3:**
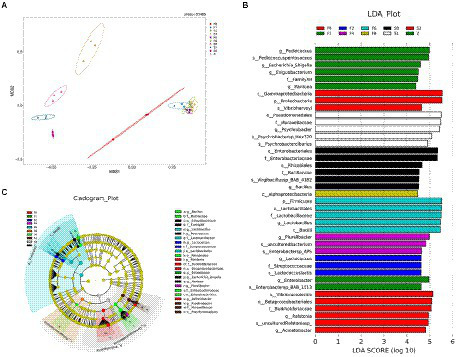
The analysis of differences of species diversity. **(A)** Two-dimensional NMDS of microbiota compositions of samples, **(B)** horizontal bar chart, **(C)** microbiota cladogram produced by LEfSe.

An NMDS (Non-metric Multidimensional Scaling analysis) ordination biplot demonstrated clear clustering of microbiota composition (Figure 3). Every point represented a sample, and the higher similarity of microbial community was, the closer the sample points were. Overall, the microbiota composition was different between fermentation and storage categories. *Zaoyu* samples on day 0 were distinct from the samples on day 1, revealing that the bacterial community changed significantly in the early fermentation. The closer distance between samples in the later fermentation stage (F2, 4, 6, 8) indicated that their bacterial community was more similar. As the fermentation system established, a dynamic and stable environment was gradually formed, leading to the similarity of microbiota composition (Shen et al., 2021).

The distinct flora of *Scomber japonicus* during different fermentation periods could be obtained based on the Lefse (Linear discriminant analysis effect size) analysis demonstrated in Figure 3B, showing those microorganisms meeting the linear discriminant analysis significance threshold of 4.0. The results revealed that the biomarkers of different samples were significantly different. The biomarker of samples of F1 was *Psychrobacter* spp., and samples of F0 featured a higher abundance of *Bacillus* spp. Compared with the controls, *zaoyu* had greater proportions of *Lactobacillus* spp. and *Lactococcus* spp. belonging to the *Lactobacillales*. These key representative bacterial taxa contributed to the differences of microbiota composition in different samples.

### Taxonomic composition of bacterial communities

**Figure 4:**
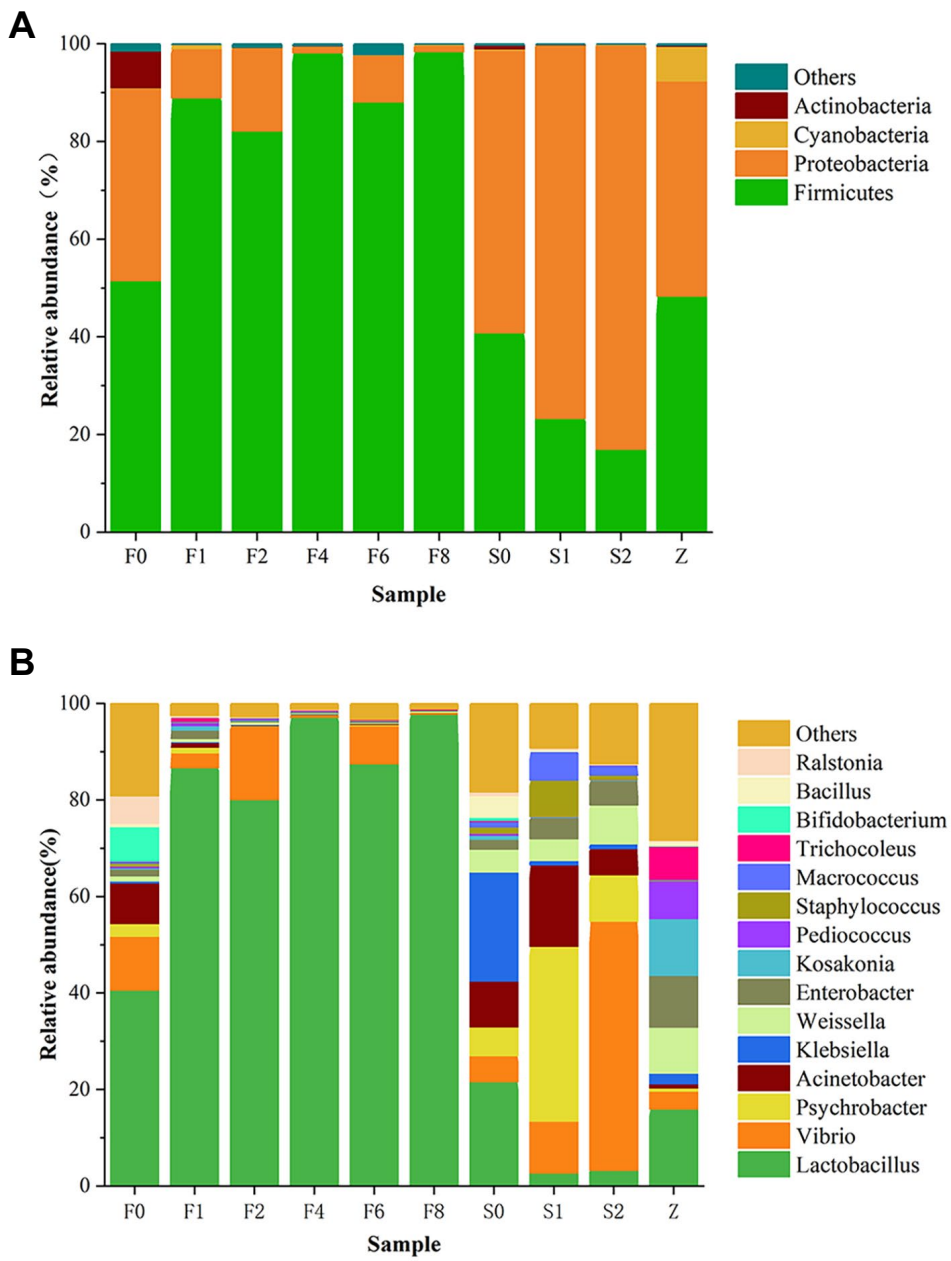
Relative abundance at the phylum level **(A)** and the genus level **(B)** of bacterial community.

To explore microbial community succession during fermentation of *Scomber japonicus*, the microbial taxonomic compositions of samples were determined at the phylum (Figure 3A) and genus (Figure 3B) levels, respectively. Among the phylum, Firmicutes and Proteobacteria possessed the highest abundance throughout the fermentation. Such results were found in other fermented seafood, like Yu-lu (Wang et al., 2018), and tilapia sausage (Li et al., 2022). As fermentation progressed, the relative abundance of Firmicutes presented an increasing trend, while the bacteria from Proteobacteria and Actinobacteria were inhibited and presented in minor percentages (Figure 4).

Analysis at genus level showed that the type of bacterial community in *zaoyu* samples was significantly lower than that of the controls, indicating that the microbial community is relatively simple during fermentation, which was in accord with the analysis of alpha diversity. *Vibrio, Psychrobacter,* and *Acinetobacter* were dominant bacterial species in the storage of *Scomber japonicus* and they were active in inducing fish spoilage, among which *Enterobacteriaceae* and *Vibrio*, two typical spoilage microorganisms playing a vital role in the formation of BAs (Kim et al., 2009), were inhibited during fermentation. The relative abundance of *Vibrio, Acinetobacter,* and *Psychrobacter* decreased rapidly along with the fermentation, whereas the relative abundance of *Lactobacillus* increased and quickly occupied the dominant position in *zaoyu*.

### Correlation between microbial community and BAs

**Figure 5:**
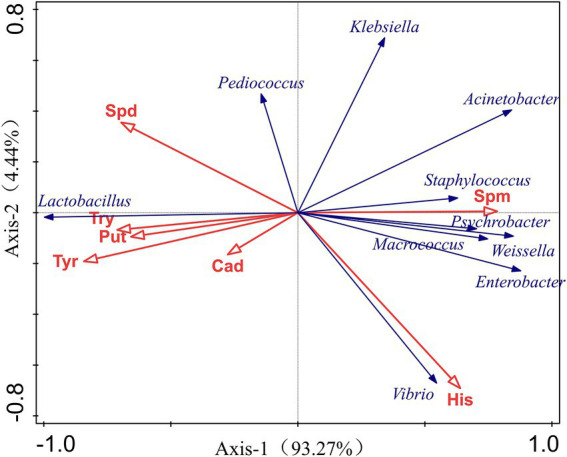
RDA for relationship between BAs and microbiota composition based on the top 10 dominant microbes at genus levels.

The RDA results demonstrated the relationship between the accumulation of BAs and bacterial community. The blue arrows expressed 7 BAs, and the red represented core microbes. The length of arrows determined the degree of importance, and the angle between the lines indicated the correlation between them. An acute angle indicated a positive correlation, while an obtuse angle indicated a negative correlation. Changes in bacterial communities were significantly correlated with the formation of different BAs. As shown in Figure 5, the accumulation of His was positively correlated with the relative abundance of *Vibrio, Enterobacter, Macrococcus,* and *Weissella,* et al., while negatively correlated with *Lactobacillus* spp. and *Pediococcus* spp. *Lactobacillus* presented positive relation to Cad, Tyr, Put and Try but negatively related to the growth of some microorganisms which was positively related to the formation of His including *Klebsiella, Acinetobacter, Vibrio,* and *Enterobacter.* These results indicated that diverse microbes like *Vibrio* spp., *Enterobacter* spp., *Macrococcus* spp., *Weissella* spp., and *Weissella* spp. belonging to gram-negative bacteria might facilitate the accumulation of His, and *Lactobacillus* may have an inhibition effect on His production by some microorganisms.

### Potential metabolic prediction By PICRUSt-KEGG

**Figure 6:**
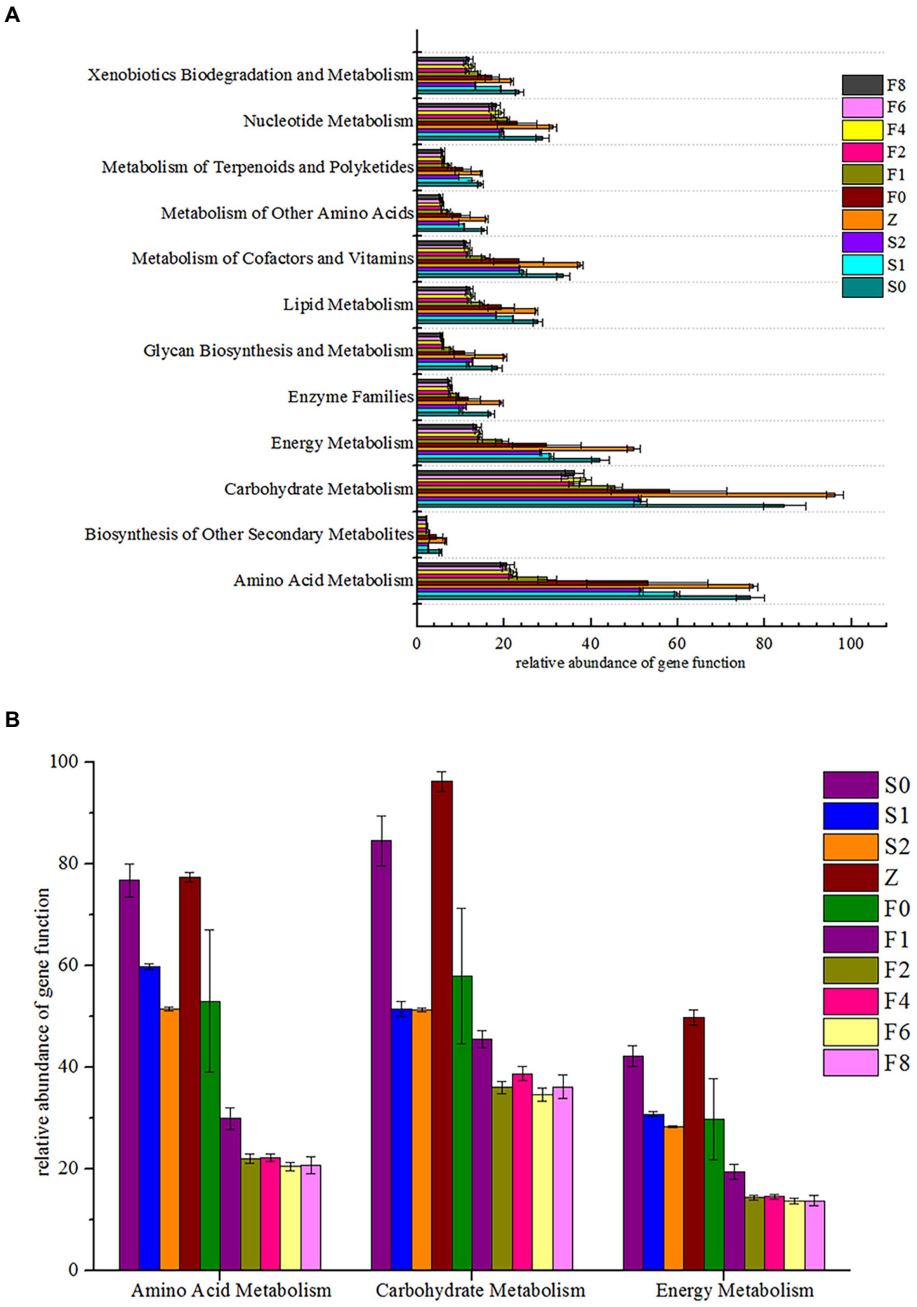
Functional genes related to metabolism. **(A)** distribution of KEGG pathways, **(B)** amino acid metabolism, carbohydrate metabolism and energy metabolism.

Since the type and abundance of metabolic pathways of microbiota determines the process of fermentation and food spoilage, the prediction of metabolic profiles may provide useful information on the potential of metabolic activities (Zhuang et al., 2020a). A total of 328 KEGG Orthology (KO) categories were obtained by assigning homologous sequences in metagenomes based on KEGG gene and pathway database. There were 12 genes related to metabolism during fermentation and storage (Figure 6A). Notably, carbohydrate metabolism presented the most enrichment among the 12 pathways, amino acid and energy metabolism were also important throughout the fermentation. Amino acid metabolism plays an important role in the spoilage of fish due to its producing carbon skeletons, especially keto acids like pyruvic acid and oxoglutarate for microbial activities and biosynthesis (Zhuang et al., 2020c).

Figure 6B showed the relative abundance of carbohydrate metabolism, amino acid metabolism, and energy metabolism of samples. Considering that all *zaoyu* samples in this study were collected in the early fermentation stage of *Scomber japonicus,* the relative gene abundance of the three metabolisms during fermentation was relatively low and changed slightly. The changes of microbiota composition may explain well why the relative enrichment levels of the metabolism-related genes decreased first, then in equilibrium during fermentation. LAB as dominant bacteria in the process of fermentation inhibited the growth of other microorganisms and caused bacteria type single.

**Figure 7:**
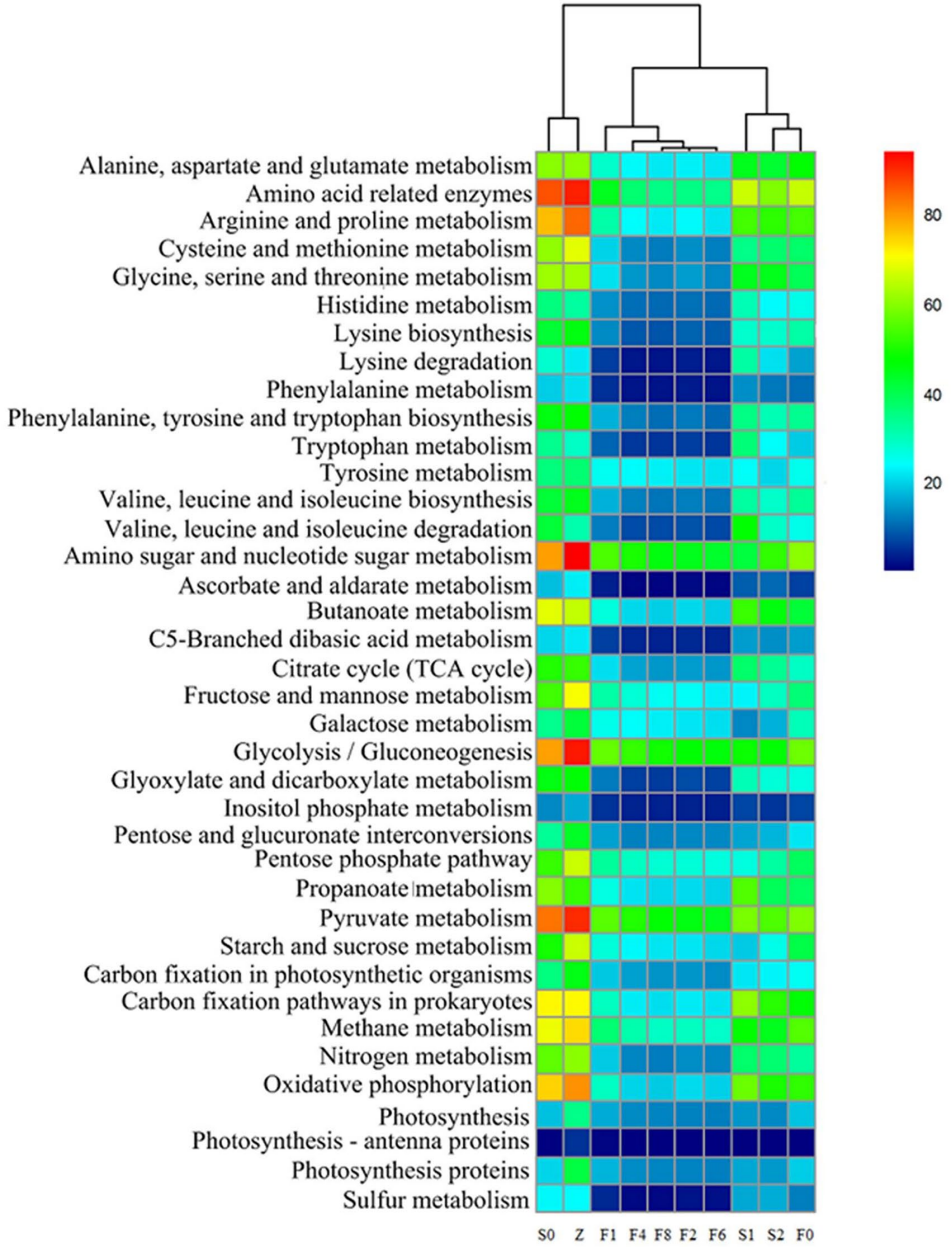
Functional prediction of amino acids, carbohydrates and energy metabolism of the microbiota.

To analyze and compare the distribution characteristics of microbial metabolism, a heatmap that represented the related gene abundances of metabolism was constructed in Figure 7, which successfully visualized the dynamic changes of various metabolisms. These main metabolic pathways presented a decreasing trend in controls, however, the changes of the metabolic features in *zaoyu* were more stable. In amino acid metabolism, histidine is a precursor substance of His, and Tyr was formed by decarboxylation of tyrosine, whereas several amino acid metabolisms including histidine, glycine, serine, and threonine metabolism were obviously inhibited during fermentation. Hence the formation of BAs in *zaoyu* was inhibited. In addition, the proteins and lipids during fermentation were mainly hydrolyzed by enzymes produced by microbial metabolism, so the relative abundance of enzyme-related genes related to amino acid metabolism was the highest in the whole process.

In carbohydrate metabolism, the main metabolic types were pyruvate metabolism, amino sugar and nucleotide sugar metabolism, as well as glycolysis. Glycolysis and pyruvate metabolism imparted a sweet taste to fermented rice, which is an essential factor affecting the flavor formation of *zaoyu*. Glycolysis is one of the most common carbohydrate metabolic pathways in bacterial communities. Pyruvate produced by glycolysis is oxidized by bacteria to acetyl-CoA for TCA cycling (Bomberg et al., 2016), and TCA cycle provides a source of many amino acid metabolisms such as lysine and glutamate.

## Discussion

The quality of *zaoyu* is closely associated with the complex metabolism of microbiota. Microbiota composition of fish flesh undergoes massive changes throughout storage and fermentation, reflected as the changes of bacterial alpha-diversity based on Shannon, Chao1, Simpson, Ace indices and the differences of dominant bacteria. It’s noteworthy that despite the highest alpha diversity in fresh samples, most species have no effect on the fermentation process due to that they originated from processing tools and environment, while these exogenous microorganisms could be inhibited under continuous fermentation and certain salt concentrations (Shen et al., 2021). Noticeable differences were observed between *zaoyu* and the controls. The bacterial diversity was relatively low in *zaoyu*, which may be attributed to the presence of dominant microorganisms such as LAB with the ability of acid tolerance. Numerous studies have shown that the presence of dominant microorganisms in aquatic products during storage or processing inhibited the growth of other microorganisms and led to a decrease in microbial diversity (Silbande et al., 2016).

The relationship between microbiota and quality of fermented seafood remained not comprehensively clear though, there has been increasing interests in forging bonds between microbiota composition and different quality indices like BAs as well as exploring the potential role of relevant microorganisms (Bover-Cid et al., 2003; Hao and Sun, 2020). Different microbes may excrete different amino acid decarboxylases to produce specific BAs, and some could also produce amine oxidases to degrade BAs in turn (Lu et al., 2018). In fermented foods, the mutual growth and interaction of LAB and yeast are universal as the growth environment is analogous (Wang et al., 2022). The majority of LAB is able to contribute to the flavor formation of fermented foods and displays some specific metabolic cross-feeding with yeast (Mukisa et al., 2017). Thus, LAB plays a solemn role in the effective fermentation of seafood. In the process of fermentation, diverse microbial interactions are thought to be more like those of an evolutionary community able to adapt to the raw material (Alekseeva et al., 2021), when microbiota have adapted to the acidic conditions, it shifts sequentially to a more acidic-tolerant group that is more adaptable to the acidic (Wang et al., 2022). *Lactobacillus* spp. occupied the dominant position throughout the whole fermentation, which is due to that it is a facultative anaerobe with the ability to survive under hypoxic and acid conditions, thus it is not restricted by the fermentation environment and maintains a constant quantity. Besides, arginine degradation has been regarded a significant source of cell energy for *Lactobacillus*, thus it could obtain energy from the formation of Put and thus keep more energetic advantages in bacterial competition (Zhuang et al., 2022). *Lactobacillus* could utilize carbohydrates in *zaoyu* to generate a large amount of lactic acid, and inhibit the growth of other spoilage bacteria like *Vibrio*, thereby reducing the content of BAs (Khouadja et al., 2017).

As the most important type of BA in the spoilage of *Scomber japonicas*, the formation of His is mainly caused by the secretion of histidine decarboxylase by some microorganisms with the ability of histidine decarboxylation to produce free histidine (Kuda et al., 2012). Some species with the ability to degrade *in vitro* Tyr and His, such as *Lactobacillus plantarum, Lactobacillus pentosus, Lactobacillus sakei, Pediococcus acidilactici*, belong to the *Lactobacillus* spp. and *Pediococcus* spp. that occupied the dominant proportions in *zaoyu* samples (Arena and Manca de Nadra, 2001; Emmanuel et al., 2010), this observation is consistent with the results of RDA analysis in this study. Therefore, it is speculated that the growth of dominant microorganisms leads to antagonism between microorganisms and thus inhibits the growth of histamine-forming bacteria. Furthermore, LAB also shows a stimulative effect on the formation of BAs. *Lactococcus, Lactobacillus, Enterococcus,* and *Pediococcus* are considered as strong producers of BAs (Ladero et al., 2012). Tyr is one of the most common and abundant BA in fermented meat products (Ruiz-Capillas and Jiménez-Colmenero, 2004), in which LAB strains of *Enterococcus faecalis, Enterococcus faecium*, and *Enterococcus durans* strains are strong tyramine-producers (Özogul and Hamed, 2017). Put could be produced through the catabolism of agmatine, a decarboxylated derivative of arginine through the agmatine deiminase (AGDI) pathway (del Rio et al., 2015). Species like *Lactobacillus brevis, Lactobacillus curvatus,* and *Enterococcus faecalis* have been reported to be able to produce putrescine *via* AGDI.

Proteobacteria was one of the predominant bacterial phyla during storage, many studies have shown that Proteobacteria is the main bacterial species in the process of seafood spoilage and plays an essential role in its quality changes (Chaiyapechara et al., 2012). It was reported that the formation of TVB-N was mainly related to *Pseudomonas* and *Enterobacteriaceae,* which were responsible for the enzymatic decarboxylation of specific amino acids (Li et al., 2019), hence it was also responsible for the accumulation of BAs. The microbial populations in the controls increased significantly and were mainly dominated by spoilage bacteria, including *Psychrobacter, Klebsiella, Vibrio,* and *Acinetobacter,* which was actively associated with the accumulation of His based on RDA analysis. The synergistic action of various microorganisms during fermentation promotes the production of Put and Cad. LAB could degrade arginine and its amino acid-derived ornithine, and then the degradation products are metabolized by *Enterobacteriaceae* to produce Put (Arena and Manca de Nadra, 2001), which may cause a symbiotic relationship between them, thus leading to a rapid increase in Put during fermentation. Kim et al. have revealed that many stains of *Enterobacter* spp. were identified as amine-forming bacteria with strong decarboxylation activity, being strong producers of Put and Cad (Kim et al., 2009). It’s noted that *Aeromonas* belonging to the *Vibrio* specie could be active in producing Cad (Zhuang et al., 2020b), which may be responsible for the relatively high content of Cad in control samples. Most Proteobacteria, including *Enterobacter*, *Salmonella*, *Vibrio,* etc. are gram-negative bacteria, indicating that the spoilage of *Scomber japonicus* is more suitable for gram-negative bacteria. *Vibrio* also may reduce trimethylamine oxides to the fishy smelling compound trimethylamine (TMA), and most strains also produce H_2_S (Lin et al., 2021). *Macrococcus caseolyticus* and *Staphylococcus sciuri*, two histamine-producing bacteria (HPB) reported before (Zhou et al., 2012), belonging to *Macrococcus* and *Staphylococcus*, respectively, were inhibited during fermentation, which could be potentially associated with the reduction of His in the whole fermentation. *Staphylococcus* has exhibited high esterase and lipase activities, and *Micrococcus* has the ability of lipolysis in fermented fish products. It could decompose proteins, free amino acids and fats fatty acids into some small molecules such as aldehydes and ketone, promoting the formation of product flavor (Lin et al., 2021).

Combined with the analysis of metabolic pathways, the metabolism of carbon and nitrogen source compounds plays an essential role during spoilage and fermentation of fish. Microbial catabolism of nitrogen-containing compounds and carbon sources could be related through biochemical reactions like amino acid transamination, which crosslinks various metabolic pathways into a complex network (Zhuang et al., 2020a). Carbohydrate metabolism and amino acid metabolism are primary metabolic pathways during storage and fermentation. The muscles of fish are rich in amino acids, providing sufficient substrate for the microorganisms. Previous studies have shown that proteins could be hydrolyzed into peptides and amino acids by microbial protease (Masniyom, 2011; Zhuang et al., 2019), then metabolized into a variety of BAs and ammonia metabolized through transamination, deamination, and decarboxylation (Biji et al., 2016). In this study, the abundance of metabolic pathways related to the formation of biogenic amines and sulfide in zaoyu was lower compared with the controls, such as phenylalanine, tyrosine and tryptophan metabolism, and these metabolisms have been reported to be associated with *Enterobacteriaceae* and *Pseudomonas* (Li et al., 2019).

Carbohydrate metabolism not only provides building blocks for the construction and assembly of complex macromolecules in cells, including nucleic acids, proteins and lipids, but is also used to generate energy required for microbial growth (Wu et al., 2021). Carbohydrates could be used as an energy source for LAB, belonging to the phylum Firmicutes which is the main microorganism of carbohydrate metabolism during fermentation (Stellato et al., 2016). LAB could compete and inhibit competitors by the rapid utilization of abundant carbohydrates with the accumulation of organic acids (Wang et al., 2022). Lactate is the main product of metabolism when the fermentable carbohydrates are abundant and generally remains the major metabolite of most LAB growing aerobically (Gänzle, 2015). A large amount of LAB produced carbon dioxide making the fermentation environment anaerobic, then synthesized pyruvic acid by anaerobic respiration. Therefore, pyruvate metabolism is the primary metabolic type of anaerobic respiration, mainly in the late stage of fermentation. Oxidative phosphorylation is a crucial type of energy metabolism in fermentation and is considered one of the most vital metabolic pathways in bacterial communities (Bomberg et al., 2016). However, further research is needed on the relationship between the metabolic pathways of exact metabolism and microbes in *zaoyu*.

## Conclusion

In this study, we characterized the complex microbiota composition during fermentation of *Scomber japonicus* using metagenomics and explored the relationships among the microbiota composition, functional metabolisms, and the formation of 8 BAs. This study revealed that the accumulation of His was inhibited and it was positively correlated with the relative abundance of *Vibrio, Enterobacter, Weissella,* et al., but negatively related to *Lactobacillus* spp. and *Pediococcus* spp., suggesting that *Lactobacillus* spp. and *Pediococcus* spp. may improve the safety of the *zaoyu* from a microbial point of view, which provided theoretical basis for the control of BAs during fermentation of *Scomber japonicus*. The predicted metabolic pathways revealed that some metabolisms related to the formation of BAs were inhibited throughout the fermentation. Nevertheless, the obtained functional profiles are merely rough functional hierarchy levels, detailed analyses of specific metabolic pathway and the roles of certain microorganism on the formation mechanisms of BAs are needed to be further elucidated.

## Data availability statement

The data presented in the study are deposited in the NCBI repository, accession number: PRJNA876092.

## Author contributions

JC performed the formal analysis, data visualization, and finished the final manuscript. HT contributed to the conception and design of the study. MZ finished the experiment. SS contributed significantly to analysis and manuscript revision. LJ helped to perform the analysis with constructive discussions. CO and HT contributed to the conception and design of the study. All authors contributed to the article and approved the submitted version.

## Funding

This work was supported by the National Key Research and Development Program of China under Grant 2019YFD0901705.

## Conflict of interest

The authors confirm that they have no known competing financial interests or personal relationships which could influence the work reported in this paper.

## Publisher’s note

All claims expressed in this article are solely those of the authors and do not necessarily represent those of their affiliated organizations, or those of the publisher, the editors and the reviewers. Any product that may be evaluated in this article, or claim that may be made by its manufacturer, is not guaranteed or endorsed by the publisher.

## Supplementary material

The Supplementary material for this article can be found online at: https://www.frontiersin.org/articles/10.3389/fmicb.2022.1030789/full#supplementary-material

## Footnotes

^1^ https://www.genescloud.cn

^2^ http://huttenhower.sph.harvard.edu/galaxy/root?tool_id=PICRUSt_normalize
